# Association between physical activity and the risk of prenatal depression in adolescents in Teresina, 2023-2024: cross-sectional study

**DOI:** 10.1590/S2237-96222025v34e20240789.en

**Published:** 2025-06-20

**Authors:** Cláudio Fernando Gomes Gonçalves, Alberto Pereira Madeiro

**Affiliations:** 1 Universidade Federal do Piauí, Programa de Pós-Graduação em Saúde e Comunidade, Teresina, PI, Brazil

**Keywords:** Depression, Pregnancy, Adolescents, Physical Exercise, Cross-Sectional Study, Depresión, Embarazo, Adolescentes, Ejercicio Físico, Estudio Transversal

## Abstract

**Objective:**

To analyze the relationship between physical activity and the risk of prenatal depression in adolescents from Teresina, Piauí, 2023-2024.

**Methods:**

Cross-sectional study with pregnant females aged 10-19 years. The Edinburgh Postnatal Depression Scale and the Physical Activity Questionnaire for Pregnant Females were used. Multiple logistic regression was used, with calculation of adjusted odds ratio (OR) and 95% confidence intervals (95%CI).

**Results:**

182 adolescents were interviewed. The prevalence of prenatal depression risk was 31.3% and sedentary lifestyle was 85.7%. Being sedentary while commuting increased the chance of depression (adjusted OR 2.40; 95%CI 1.01; 5.68; p-value 0.046), while practicing physical exercise (adjusted OR 0.22; 95%CI 0.05; 0.94; p-value 0.041) and having light energy expenditure in daily household chores (adjusted OR 0.26; 95%CI 0.07; 0.98; p-value 0.047) decreased the risk of depression.

**Conclusion:**

The prevalence of prenatal depression and sedentary lifestyle during pregnancy was high. Exercising and being active in household chores reduced the risk of prenatal depression among pregnant adolescents.

## Resumen

**Objetivo:**

Analizar la relación entre la actividad física y el riesgo de depresión prenatal en adolescentes de Teresina, Piauí, 2023-2024.

**Métodos:**

Estudio transversal con mujeres embarazadas de 10 a 19 años. Se utilizó la Escala de Depresión Postnatal de Edimburgo y el Cuestionario de Actividad Física para Embarazadas. Se utilizó regresión logística múltiple, con cálculo de *odds ratio* (OR) ajustado e intervalos de confianza del 95% (IC95%).

**Resultados:**

Se entrevistaron 182 adolescentes. La prevalencia de riesgo de depresión prenatal fue de 31,3% y de sedentarismo de 85,7%. El sedentarismo durante el desplazamiento aumentó la probabilidad de depresión (OR 2,40; IC95% 1,01; 5,68; p-valor 0,046), mientras que la práctica de ejercicio físico (OR 0,22; IC95% 0,05; 0,94; p-valor 0,041) y tener un gasto energético ligero en actividades domésticas (OR 0,26; IC95% 0,07; 0,98; p-valor 0,047) disminuyó la probabilidad de depresión.

**Conclusión:**

La prevalencia de depresión prenatal y sedentarismo durante el embarazo fue alta. Hacer ejercicio y participar activamente en las tareas domésticas redujo el riesgo de depresión prenatal entre los adolescentes.

**Ethical aspects**

This research respected ethical principles, having obtained the following approval data::

Research Ethics Committee: Universidade Federal do Piauí

Opinion number: 5.890.935

Approval date: 13/2/2023

Certificate of Submission for Ethical Appraisal: 66480823.7.0000.5214

Informed Consent Form: Obtained from all participants prior to collection.

## Introduction

Depression is one of the main causes of illness worldwide, with increasing incidence in several countries (1). In Brazil, the 2019 National Health Survey revealed that approximately 16.3 million (10.2%) people suffer with depression, with a higher prevalence among females (14.7%) (2). People with this disorder commonly experience a strong sense of hopelessness, depressed mood, irritability, low self-esteem, lack of energy and loss of interest and pleasure in life, with a negative impact on work capacity, academic performance and daily activities (3).

Women of reproductive age are more vulnerable to mental health disorders, especially during pregnancy. During this period, multiple physiological and emotional transformations occur, which favor the acquisition of mental disorders (4). The factors that contribute to the development of depression during pregnancy are well known, such as psychological history, unplanned pregnancy, being a single mother and/or adolescent mother, lack of family support and financial difficulties (4,5). In Brazil, the prevalence of prenatal depression varies between 11% and 16% (6,7), exceeding the global average of 10% to 13% (5).

Women who suffer from prenatal depression are more likely to adopt unhealthy behaviors, including poor nutrition, alcohol and tobacco abuse, lack of physical activity, and lack of prenatal care (4). Problems related to depression can also result in adverse health outcomes for the woman and the fetus, particularly pre-eclampsia, fetal growth retardation and premature birth (8).Pregnant adolescents are at greater risk of psychological problems, due to emotional immaturity and inadequate preparation for motherhood (9).In addition to health risks, teenage pregnancy can trigger biopsychosocial and economic problems, directly affecting the personal and professional future of these young females (10).

Despite advances in knowledge about mental health, prenatal depression is often overlooked and confused with common emotional changes in pregnancy (9). The practice of physical activity appears as a safe and accessible alternative for the non-pharmacological treatment of depressive symptoms (11), in addition to being able to reduce gestational diabetes, pre-eclampsia and low back pain (11). It is recommended that pregnant women practice at least 150 minutes of aerobic physical activity per week, lasting 20 to 30 minutes and at moderate intensity on most or all days of the week (12).

In 2019, 61.8% of adolescents were insufficiently active and only 28.1% showed active patterns of exercise (13). These numbers are even higher among pregnant adolescents, and 84.2% of them may exhibit sedentary behavior (14). Although physical activity can promote numerous health benefits for pregnant females, little is known about its influence on prenatal depression in adolescents. Most of the available studies have been limited to studying the effects of physical activity during pregnancy and prenatal depression separately (4,14,15). This study aimed to analyze the relationship between physical activity and prenatal depression in adolescents from Teresina, Piauí.

## Methods

### Study design

This is a cross-sectional study with pregnant adolescents from Teresina, Piauí, carried out between April 2023 and March 2024. 

### Context

Teresina, capital of Piauí, had 871 thousand inhabitants in 2021. In 2022, the municipality had 569 registered health institutions. Of these, 90 were Basic Health Units that had 264 Family Health Strategy teams. In 2020, the municipality’s primary health care service monitored 1,439 pregnant females aged 10 to 19, with at least 1 prenatal consultation carried out (16).

### Participants

The population of this study was composed of pregnant females between 10 and 19 years old who received prenatal care through the Unified Health System. Women with obstetric conditions that prevented them from performing physical activities during the current pregnancy were excluded. Such conditions include placenta previa, preterm labor, premature rupture of membranes, pre-eclampsia or pregnancy-induced hypertension, severe anemia, persistent bleeding in the second or third trimester, cervical incompetence, multiples gestation with risk of preterm birth, restrictive lung disease, and hemodynamically significant heart disease (12).

### Sample size

The sample size was calculated taking into account the number of live births among adolescents aged 10 to 19 in Teresina in 2020 and the prevalence of prenatal depression of 16% in Brazilian females (6). Thus, after applying the 95% confidence interval, 5% error margin and 10% increase for losses, a minimum sample of 182 participants was estimated.

### Data source/bias control

Data collection took place in two stages. First, Basic Health Units were drawn from each area of the city (Center/North, South, East/Southeast) until the minimum sample size was reached. In the second stage, the adolescents were approached either before or after the prenatal consultation and invited to participate in the research. When there was a refusal or there were no adolescents attending prenatal care, the institutions were replaced by others in the same area. In cases where the adolescents did not accept the invitation or were unable to participate, another adolescent was invited as a replacement. All interviews were conducted by a trained researcher and took place at the health unit where the adolescent received prenatal care. A pilot survey was carried out with 10 participants to assess understanding of the questions and, after adjustments, the pre-test responses were discarded. 

### Variables

Three instruments were used. The first contained questions about variable such as: a) sociodemographic: age group (in years: 10-14; 15-19); race/skin color (white; yellow (Asian); brown; black; indigenous); type of school (public; private); education (<elementary school; ≥elementary school); mother’s education (no education; incomplete or complete elementary school; incomplete or complete high school; incomplete and complete higher education); school dropout (due to pregnancy: yes; no); lives with mother and/or father (yes; no); working (yes; no); marital status (with partner; without partner); b) behavioral: alcohol consumption (during pregnancy: yes; no); cigarette smoking (during pregnancy: yes; no); illicit drug use (during pregnancy: yes; no); depressive disorder (prior to pregnancy: yes; no); anxiety disorder (prior to pregnancy: yes; no); suicidal ideation (prior to pregnancy: yes; no); c) physical activity habits: type of physical exercise (usually practiced: walking; swimming; cycling; aerobic gymnastics; strength training; yoga or Pilates; other); physical exercise recommended by a health professional (yes; no); physical exercise with the supervision of a physical education professional (yes; no); frequency of physical activity (times per week: 1; 2-3; more than 3; not applicable); self-perceived level of physical activity (inactive; slightly active; active); d) obstetric: number of pregnancies (including the current one: 1; ≥2); number of abortions (0; ≥1); number of births (0; ≥1); number of prenatal consultations (<6; ≥6); planned pregnancy (yes; no); feelings towards unplanned pregnancy (wanted; unwanted; not applicable); e) marital relationship: duration of partnership (<1 year; >1 year; not applicable); communication with partner (good; bad; not applicable); fights between the couple (no; 01 time/month; >1 time/month; not applicable). 

The second instrument investigated the risk of prenatal depression, using the Edinburgh Postnatal Depression Scale, already validated in Brazil (17). This scale consists of 10 questions, used to screen for depressive symptoms in pregnant or postpartum women. Each question has 4 alternatives scored from 0 to 3 according to the intensity of the symptoms, with scores greater than or equal to 13 being considered as a risk of developing depression (18). 

Information on physical activity habits was obtained from the Physical Activity Questionnaire for Pregnant Women, aimed at the population of pregnant females, which investigates the relationship between duration, frequency, type of physical activity and energy expenditure in 33 activities (12 domestic, 5 occupational, 9 sports/exercise, 3 transportation/commuting and 3 inactivity) in the last three months. Each question has energy expenditure values equivalent to its intensity and, to obtain the weekly energy average, the individual energy consumption of the activities was multiplied by the number of minutes each of them was performed (19). With this result, the following were evaluated: a) level of physical activity: sedentary (value less than 1.5); light (1.5-3.0); moderate (3.0-6.0); vigorous (greater than 6); b) level of physical activity by domain: household tasks; occupational; caring for people; sports/exercise; leisure; commuting. 

### Statistical methods

The data were analyzed descriptively using absolute (n) and relative (%) frequencies with the *Statistical Package for the Social Science* software. Pearson’s chi-square test/Fisher’s exact test was used to assess the association between the outcome of (prenatal depression) and physical activity, in addition to other independent variables. Crude odds ratio (crude OR) and 95% confidence intervals (95% CI) were calculated. All variables that showed p-value <0.20 were included in the multivariate analysis using multiple logistic regression, with calculation of adjusted odds ratio (adjusted OR) and 95%CI. The significance level considered was 5%. The final regression model was adjusted for the variables of age group, education, marital status, unplanned pregnancy, history of depression and suicidal ideation, using the Enter method. 

## Results

182 pregnant adolescents were interviewed. Table 1 shows that the most of them were between 15 and 19 years old (95.6%), were brown skinned (58.8%) and had completed elementary education (85.2%). The majority did not live with their parents (54.4%), were in a marital partnership (80.2%) and did not report any working employment (86.3%). Regarding maternal education, the majority studied up to elementary school (40.7%). All adolescents went to public schools (100%) and almost a third (30.2%) reported dropping out of school due to pregnancy.

**Table 1:** Sociodemographic characteristics of pregnant adolescents. Teresina, 2023-2024 (n=182)

Variables	n (%)
**Age group** (years)	
10-14	8 (4.4)
15-19	174 (95.6)
**Race/skin color**	
White	28 (15.4)
Black	34 (18.7)
Yellow (Asian)	10 (5.5)
Brown	107 (58.8)
Indigenous	2 (1.1)
Not specified	1 (0.5)
Education	
<Elementary education	27 (14.8)
≥Elementary school	155 (85.2)
**Lives with mother and/or father**	
Yes	83 (45.6)
No	99 (54.4)
**Marital status**	
With a partner	146 (80.2)
Without a partner	36 (19.8)
**Maternal education**	
Uneducated	6 (3.3)
Elementary education	74 (40.7)
High school	73 (40.1)
Higher education	8 (4.4)
Don’t know/didn’t want to specify	21 (11.5)
**Type of school**	
Public	182 (100.0)
**Working employment**	
Yes	25 (13.7)
No	157 (86.3)
**School dropout**	
Yes	55 (30.2)
No	127 (69.8)

The majority were pregnant for the first time (75.3%), without previous births (84.1%), without abortions (87.4%) and with 6 or more prenatal consultations (55.5%). The report of unplanned pregnancy was the most common (80.2%), however, among these, the majority reported that the pregnancy was desired (89.7%). There was a predominance of pregnant females who had been in a relationship for one year or more (74.7%) and who often had arguments with their partner (62.3%). Almost all reported having good communication with their partner (97.3%). During the pregnancy, the majority reported not consuming alcoholic beverages (90.1%), not smoking (97.8%) and not using illicit drugs (98.5%). Prior to pregnancy, there were reports of depression (10.4%), anxiety (41.8%) and suicidal ideation (20.9%). The risk of developing prenatal depression was 31.3% (Table 2).

**Table 2:** Obstetric, behavioral and marital relationship characteristics of pregnant adolescents. Teresina, 2023-2024 (n=182)

Variables	n (%)
**Number of pregnancies**	
1	137 (75.3)
≥2	45 (24.7)
**Number of previous births**	
0	153 (84.1)
≥1	29 (15.9)
**Number of abortions**	
0	159 (87.4)
≥1	23 (12.6)
**Number of prenatal consultations**	
<6	81 (44.5)
≥6	101 (55.5)
**Planned pregnancy**	
Yes	36 (19.8)
No	146 (80.2)
**Feelings about pregnancy** (unplanned) (n=146)	
Wanted	131 (89.7)
Unwanted	15 (10.3)
**Duration of partnership** (n=146)	
Less than a year	37 (25.3)
A year or more	109 (74.7)
**Fights between the couple** (n=146)	
Don’t usually fight	55 (37.7)
Once a month	51 (34.9)
More than once a month	40 (27.4)
**Communication with partner** (n=146)	
Good	142 (97.3)
Bad	4 (2.7)
**Alcohol use**	
Yes	18 (9.9)
No	164 (90.1)
**Cigarette use**	
Yes	4 (2.2)
No	178 (97.8)
**Use of illicit drugs**	
Yes	3 (1.5)
No	179 (98.5)
Depressive disorder	
Yes	19 (10.4)
No	163 (89.6)
**Anxiety disorder**	
Yes	76 (41.8)
No	106 (58.2)
**Suicidal ideation**	
Yes	38 (20.9)
No	144 (79.1)
**Risk of prenatal depression** (score)	
<13	125 (68.7)
≥13	57 (31.3)

Most of them did not practice any physical exercise (67.6%). Among those who practiced it, there was a predominance of those who exercised 2 to 3 times a week (57.6%), without supervision from a physical education professional (91.5%) and without recommendation from a health professional (78.0%), with walking being the most frequent exercise (83.0%). Although the majority considered themselves slightly active/active (73.6%), the score obtained in the Physical Activity Questionnaire for Pregnant Women revealed that most of them were classified as sedentary (85.7%) (Table 3).

**Table 3:** Physical activity habits of pregnant adolescents. Teresina, 2023-2024 (n=182)

Variables	n (%)
**Practice of physical exercise**	
Yes	59 (32.4)
No	123 (67.6)
**Frequency of physical exercise** (**times per week**) (n=59)	
1	14 (23.7)
2 to 3	34 (57.6)
3 or more	11 (18.7)
**Exercise recommended by health professional** (n=59)	
Yes	13 (22.0)
No	46 (78.0)
**Exercise supervised by a physical education professional** (n=59)	
Yes	5 (8.5)
No	54 (91.5)
**Type of exercise** (n=59)^a^	
Walking	49 (83.0)
Cycling/aerobics/dancing	10 (16.9)
Strength training/bodybuilding/crossfit	7 (11.8)
Yoga/pilates	2 (3.4)
Swimming	1 (1.7)
Other	3 (5.0)
**Self-perception of physical activity level**	
Sedentary	48 (26.4)
Slightly active	71 (39.0)
Active	63 (34.6)
**Physical activity level**	
Sedentary	156 (85.7)
Light	25 (13.7)
Moderate	1 (0.6)

^a^More than one option could be chosen.

The level of physical activity by domains can be seen in Table 4. A sedentary lifestyle was higher in the domains of sport/exercise (100%), commuting (85.7%) and caring for people (74.2%). The domain corresponding to domestic tasks presented the highest energy expenditure in the light exercise classification (49.0%) and leisure in the moderate exercise classification (18.7%). The domains of working employment (3.9%) and domestic tasks (1.1%) were the only ones that had participants in the vigorous classification.

**Table 4:** Level of physical activity by domains of the Physical Activity Questionnaire for Pregnant Women. Teresina, 2023-2024 (n=182)

Physical activity level	Sedentary n (%)	Light n (%)	Moderate n (%)	Vigorous n (%)
Household chores	82 (45.0)	89 (49.0)	11 (6.0)	-
Taking care of people	135 (74.2)	23 (12.6)	22 (12.1)	2 (1.1)
At work	129 (70.9)	27 (14.8)	19 (10.4)	7 (3.9)
Sport/exercise	182 (100)	-	-	-
Commuting	156 (85.7)	22 (12.1)	4 (2.2)	-
Leisure	62 (34.1)	86 (47.2)	34 (18.7)	-

After adjustments, the multivariate analysis identified that pregnant adolescents who performed physical exercise (adjusted OR 0.22; 95%CI 0.05; 0.94; p-value 0.041) and who had light energy expenditure in household chores (adjusted OR 0.26; 95%CI 0.07; 0.98; p-value 0.047) showed a lower chance of developing prenatal depression. However, being sedentary when commuting (adjusted OR 2.40; 95%CI 1.01; 5.68; p-value 0.046) increased the chance of prenatal depression (Table 5).

**Table 5:** Crude odds ratio (crude OR) and adjusted odds ratio (adjusted OR) and 95% confidence intervals (95%CI) of antenatal depression and physical activity. Teresina, 2023-2024

Variables	Prenatal depression			
	Not adjusted		Adjusted^a^	
	Crude OR (95%CI)	p-value	Adjusted OR (95%CI)	p-value
**Practice of physical exercise**				
Yes	0.58 (0.28; 0.97)	0.026	0.22 (0.05; 0.94)	0.041
No	1.00		1.00	
**Weekly frequency of physical exercise**				
Once a week	1.00		-	
2 to 3 times	0.90 (0.22; 3.60)	0.882	-	-
More than 3 times	0.25 (0.02; 2.65)	0.250	-	-
**Exercise recommended by a health professional**				
Yes	4.07 (1.08; 15.40)	0.031	1.87 (0.91; 3.87)	0.090
No	1.00		1.00	
**Type of exercise**				
Walking	0.34 (0.07; 1.53)	0.159	0.29 (0.06; 1.37)	0.117
Walking and other type	0.56 (0.09; 3.52)	0.538	0.50 (0.08; 3.21)	0.463
Other	1.00		1.00	
**Self-perception of physical activity level**				
Sedentary	1.20 (0.55; 2.63)	0.649	-	-
Slightly active	0.68 (0.32; 1.44)	0.538	-	-
Active	1.00			
**Physical activity level**				
Sedentary	1.03 (0.42; 2.53)	0.950	-	-
Mild/moderate	1.00			
**Level of physical activity** (**household chores**)				
Sedentary	0.39 (0.11; 1.38)	0.144	0.44 (0.12; 1.56)	0.206
Light	0.33 (0.09; 1.16)	0.084	0.26 (0.07; 0.98)	0.047
Moderate	1.00		1.00	
**Physical activity level** (**caring for people**)				
Sedentary	1.66 (0.58; 4.74)	0.346	1.60 (0.55; 4.64)	0.396
Light	3.48 (0.97; 2.53)	0.056	3.00 (0.83; 11.24)	0.092
Moderate/vigorous	1.00		1.00	
**Level of physical activity** (**at work**)				
Sedentary	2.70 (0.31; 23.14)	0.366	-	-
Light	2.53 (0.26; 24.51)	0.424	-	-
Moderate	1.00			
Vigorous	4.36 (0.43; 43.72)	0.210	-	-
**Physical activity level** (commuting)				
Sedentary	0.39 (0.17; 0.91)	0.027	2.40 (1.01; 5.68)	0.046
Mild/moderate	1.00		1.00	
**Level of physical activity** (leisure)				
Sedentary	1.52 (0.62; 3.72)	0.364	-	-
Light	0.87 (0.62; 3.72)	0.768	-	-
Moderate	1.00			

^a^Model adjusted for age group, education, marital status, unplanned pregnancy, history of depression, suicidal ideation.

## Discussion

The data showed that about a third of pregnant adolescents were at risk of developing prenatal depression. The majority did not performed physical exercise and, despite considering themselves active during pregnancy, the majority were classified as sedentary. Most health professionals did not recommend physical activity during pregnancy and, among those adolescents who exercised, a small proportion did so under the guidance of a physical education professional. Walking was the predominant type of exercise, with greater time spent on household and leisure tasks. There was an increased chance of prenatal depression in those who were considered sedentary during commuting. On the other hand, performing physical exercise and having light energy expenditure in household chores determined a lower chance of developing prenatal depression.

At least three limitations must be considered in this study. First, despite the use of validated instruments, self-reports may have been affected by recall bias. Another issue to be mentioned is the short period assessed by the Questionnaire (3 months), which is insufficient to assess the entire pregnancy, leading to the need for longitudinal studies. Finally, the participants were pregnant females who visited public health services, which limits the ability for results generalization. On the other hand, one of the relevant points of this study was to investigate the population of pregnant adolescents, doubly vulnerable and with little evidence, which presented high rates of physical inactivity and risk of prenatal depression. 

The prevalence of prenatal depression found in this research was higher than that observed in other countries. Approximately 20% of women experience some form of mental health episode during pregnancy (1), with global rates of prenatal depression ranging from 15% to 65% and higher prevalence in low- and middle-income countries (20). In Brazil, the prevalence varies between 11.4% and 26.8% (7,21). However, when the sample is composed only of pregnant adolescents, the rates can be even higher, as observed in Pelotas, Rio Grande do Sul (29.2%) (22) and in Bandeirantes, Paraná (49.1%) (23).

Differences in outcomes may be attributed to socioeconomic and cultural variables, such as social support and stress levels associated with different socioeconomic contexts (24). Regional socioeconomic disparities in education, health and social support may influence these results (7). In regions with less access to mental health services and a higher incidence of poverty, the prevalence of prenatal depression tends to be higher (25). Furthermore, the assessment instrument used can influence the results. In this study, the Edinburgh Postnatal Depression Scale was used, which generally shows lower levels of depression (5).

Physical inactivity was predominant among pregnant adolescents, with the majority classified as sedentary. This finding is consistent with studies demonstrating low levels of physical activity among pregnant women globally (26,27). Despite the lack of specific information on physical activity in pregnant adolescents, Brazilian data show a reduced level of physical activity during pregnancy in this population (14,28). In 2010, a study carried out in Coari, Amazonas, assessed the perception of sedentary lifestyle among pregnant adolescents, identifying that 97% of them claimed not to exercise during pregnancy (28). 

Several factors may contribute to the high prevalence of physical inactivity and sedentary lifestyle in this population. Lack of knowledge and guidance on the importance of physical activity during pregnancy may lead these young women to underestimate the benefits of regular exercise (11,26,27). Socioeconomic barriers, such as lack of access to safe places to practice physical activities and lack of financial resources to participate in supervised exercise programs, also play a significant role (29). Another limiting factor is the fear of harming the pregnancy, which can be exacerbated by the lack of adequate guidance from health professionals (30). The presence of depressive and anxious symptoms can also reduce the motivation and energy needed to practice physical activities (3,4). In combination, these factors create an unfavorable environment for the adoption of an active lifestyle during pregnancy among adolescents.

In the current study, most professionals did not recommend physical activity during pregnancy, according to the adolescents’ reports. This data deserves to be highlighted, because it corroborates other research that identified the low recommendation for physical exercise. A 2019 study of 1,082 pregnant women in South Africa found that 64.7% of them did not receive any advice on physical activity during their antenatal visits (29). Similarly, a study cohort of 118 Portuguese pregnant women between 2009 and 2011 observed that approximately one third of health professionals had not recommended physical activity during pregnancy and, among those who did, the majority recommended it only in the second semester of pregnancy (31).

Most adolescents who performed physical activity did so insufficiently and without the supervision of physical education professionals, which may reflect difficulties in access or a lack of awareness about the importance of this specialized support. Although it is known that regular physical exercise promotes several health benefits for pregnant women (11,26), experts warn of necessary precautions, such as avoiding activities that involve physical contact, jumping, isometric exercises and diving (12). Pregnant females with certain health conditions, such as heart or respiratory disease, severe anemia, multiples pregnancy, placenta previa, pre-eclampsia and bleeding, should not perform physical exercises (12). Follow-up by a physical education professional is essential to develop personalized and safe exercise programs, prevent injuries and complications, and provide motivational and psychological support. 

Another aspect that drew attention in this study was that, even among adolescents who considered themselves slightly active and active, the majority were classified as sedentary. This paradox may be attributed to a misperception about what constitutes sufficient physical activity to achieve health benefits, suggesting that many individuals underestimate the activity levels needed to maintain mental and physical health (11,27). Pregnant women often consider their daily activities to be sufficient to remain physically active, despite not reaching the recommended levels of physical activity (26,29). The type of physical exercise most practiced by pregnant adolescents was walking, with a predominance of sessions 2 to 3 times a week. The recurrence of this practice is possibly due to its accessibility and ease of implementation (26,27,29). However, the lack of variety and low intensity of activities are reflected in the high rate of sedentary lifestyle identified by the Physical Activity Questionnaire for Pregnant Women in this study.

Most of the weekly time of the pregnant women in this study was spent on leisure and domestic activities, with a higher prevalence of sedentary lifestyle in the domains of sport/exercise, commuting and caring for people. Similarly, a 2019 investigation with 1,279 pregnant women from Montes Claros, Minas Gerais, identified that in the categories of caring for people, sports and commuting, respectively, 89.0%, 93.1% and 93.2% of the interviewees were classified as physically inactive (32). These numbers reflect a lifestyle that lacks regular physical movement, especially in structured physical activities or sports, which is worrying, as regular exercise during pregnancy is associated with significant benefits for both the woman and the fetus (11).

Adolescents classified as sedentary when commuting were more likely to develop prenatal depression compared to those with light or moderate levels of physical activity in this domain. This finding is consistent with other studies that have shown that active commuting, such as walking or cycling, can provide significant psychological benefits, including reduced risk of depression, likely through exposure to the outdoors and social interaction (33,34). Adolescents who performed physical exercise and those with light energy expenditure on household chores had a lower chance of developing prenatal depression. On the one hand, because it is stressful especially for women, domestic physical activity may have no effect or even increase the risk of depression (35). On the other hand, even at light levels, this type of physical activity has a protective effect against depression during pregnancy (36,37).

Even though it is still uncertain which type of physical activity is most effective in mitigating the effects of prenatal and postpartum depression, the World Health Organization recommends that all pregnant women without contraindications perform at least 150 minutes of moderate-intensity aerobic exercise per week, combined with strength training and relaxation activities (38). Both aerobic and group exercises, activities in aquatic environments and those chosen by the pregnant woman can alleviate the symptoms of prenatal depression (36,37,39). However, towards the end of pregnancy, women tend to feel physically tired and light physical activities, such as meditation and body relaxation, also have a positive impact on the mental health of pregnant women (37,39).

Considering the positive effect of physical activity on the occurrence of prenatal depression, the results of the present study may corroborate the implementation of interventions that encourage the practice of physical activity among adolescents. Future research should explore the types, intensity, and duration of physical activity that are most appropriate and effective for depression severity levels. One of the challenges concerns the need for personalized interventions that take into account not only the recommendation of health professionals, but also the preferences and individual context of adolescents to promote physical activity. Considering the vulnerability of this population, health promotion strategies that integrate emotional and motivational support can be especially beneficial. From a public health perspective, the inclusion and performance of physical education professionals in primary care expands the offer of multidisciplinary health promotion, through body practices and systematized physical activity. It is possible that such actions will result in a reduction in the high prevalence of prenatal depression and the negative outcomes related to it.

## Data Availability

Access to the database and the analysis codes, methods and other materials used in the research may be requested from the coordinating author of the research or the corresponding author.
