# Supplementary material for: Association between physical activity and the risk of prenatal depression in adolescents in Teresina, 2023-2024: cross-sectional study
Source: Epidemiol Serv Saude. 2025 Jun 20;34:e20240789. doi: 10.1590/S2237-96222025v34e20240789.en (PMC12204066; doi:10.1590/S2237-96222025v34e20240789.en)
Supplement: Translation

# Translation: Associação entre atividade física e risco de depressão pré-natal em adolescentes em Teresina, 2023-2024: estudo transversal

**DOI:** 10.1590/S2237-96222025v34e20240789.pt

**Authors:** Cláudio Fernando Gomes Gonçalves, Alberto Pereira Madeiro

**Affiliations:** 1 Universidade Federal do Piauí, Programa de Pós-Graduação em Saúde e Comunidade, Teresina, PI, Brasil

**Keywords:** Depressão, Gravidez, Adolescentes, Exercício Físico, Estudo Transversal

## Abstract

**Objetivo:**

Analisar a relação entre atividade física e o risco de depressão pré-natal em adolescentes de Teresina, Piauí, 2023-2024.

**Métodos:**

Estudo transversal, com gestantes de 10-19 anos. Foram utilizados a Escala de Depressão Pós-Parto de Edimburgo e o Questionário de Atividade Física para Gestantes. Empregou-se regressão logística múltipla, com cálculo de razão de chances ajustada (*odds ratio*, OR) e intervalos de confiança de 95% (IC95%).

**Resultados:**

Foram entrevistadas 182 adolescentes. A prevalência do risco de depressão pré-natal foi de 31,3% e de sedentarismo de 85,7%. Ser sedentária no deslocamento aumentou a chance de depressão (OR 2,40; IC95% 1,01; 5,68; p-valor 0,046), enquanto praticar exercício físico (OR 0,22; IC95% 0,05; 0,94; p-valor 0,041) e ter gasto energético leve em atividade domésticas (OR 0,26; IC95% 0,07; 0,98; p-valor 0,047) diminuíram a chance de depressão.

**Conclusão:**

As prevalências do risco de depressão pré-natal e de sedentarismo durante a gravidez foram elevadas. Praticar exercício físico e ser ativa em atividades domésticas diminuíram o risco de depressão pré-natal entre adolescentes.

**Aspectos éticos**

A presente pesquisa respeitou os princípios éticos, obtendo os seguintes dados de aprovação: :

Comitê de ética em pesquisa: Universidade Federal do Piauí

Número do parecer: 5.890.935

Data de aprovação: 13/2/2023

Certificado de apresentação de apreciação ética: 66480823.7.0000.5214

Registro do consentimento livre e esclarecido: Obtido de todos os participantes antes da coleta.

## Introdução

A depressão é uma das principais causas de doença em todo o mundo, com incidência crescente em diversos países (1). No Brasil, a Pesquisa Nacional de Saúde de 2019 revelou que há cerca de 16,3 milhões (10,2%) de pessoas com depressão, sendo a prevalência maior entre o sexo feminino (14,7%) (2). Pessoas com esse distúrbio apresentam comumente forte sentimento de desesperança, humor deprimido, irritabilidade, baixa autoestima, falta de energia, perda de interesse e de prazer pela vida, com impacto negativo na capacidade laboral, no desempenho acadêmico e nas atividades diárias (3).

Mulheres em idade reprodutiva são mais vulneráveis aos distúrbios de saúde mental, sobretudo durante a gravidez. Nesse período, ocorrem múltiplas transformações de caráter fisiológico e emocional, o que favorece a aquisição de transtornos mentais (4). São bem conhecidos os fatores que contribuem para o desenvolvimento da depressão durante a gravidez, como os antecedentes psicológicos, a gravidez não planejada, ser mãe solteira e/ou mãe adolescente, a falta de suporte familiar e dificuldades financeiras (4,5). No Brasil, a prevalência de depressão pré-natal varia entre 11% e 16% (6,7), superando a média global de 10% a 13% (5).

As mulheres que sofrem com depressão pré-natal são mais propícias a adotarem atitudes não saudáveis, incluindo má nutrição, uso abusivo de álcool e tabaco, inatividade física e falta de cuidados pré-natais (4). Problemas relacionados à depressão também podem resultar em desfechos adversos para saúde da mulher e do feto, com destaque para a pré-eclâmpsia, o retardo no crescimento fetal e o parto prematuro (8). Grávidas adolescentes apresentam maior risco de problemas psicológicos, tanto por imaturidade emocional como pela preparação inadequada para a maternidade (9). Além dos riscos à saúde, a gestação na adolescência pode desencadear problemas biopsicossociais e econômicos, afetando diretamente o futuro pessoal e profissional dessas jovens (10).

Apesar dos avanços no conhecimento sobre saúde mental, a depressão pré-natal é frequentemente negligenciada, muitas vezes confundida com alterações emocionais comuns na gravidez (9). A prática de atividade física surge como alternativa segura e acessível para o tratamento não-farmacológico dos sintomas depressivos (11), além de pode reduzir diabetes gestacional, pré-eclâmpsia e lombalgia (11). Recomenda-se que mulheres grávidas pratiquem pelo menos 150 minutos de atividade física aeróbica por semana, com duração de 20 a 30 minutos e intensidade moderada na maioria ou em todos os dias da semana (12).

Em 2019, 61,8% dos adolescentes eram insuficientemente ativos e apenas 28,1% apresentaram padrões ativos (13). Esses números são ainda maiores entre adolescentes grávidas, em que 84,2% delas podem apresentar comportamento sedentário (14). Embora a atividade física seja capaz de promover inúmeros benefícios à saúde das gestantes, pouco se sabe sobre sua influência na depressão pré-natal em adolescentes. A maioria dos estudos disponíveis têm se limitado a estudar os efeitos da atividade física na gravidez e a depressão pré-natal separadamente (4,14,15). Este estudo objetivou analisar a relação entre a atividade física e a depressão pré-natal em adolescentes de Teresina, Piauí.

## Métodos

### Delineamento

Trata-se de estudo transversal, com gestantes adolescentes de Teresina, Piauí, realizado entre abril de 2023 e março de 2024. 

### Contexto

Teresina, capital do Piauí, possuía 871 mil habitantes em 2021. No ano de 2022, o município apresentava 569 instituições de saúde cadastradas. Destas, 90 eram unidades básicas de saúde que contavam com 264 equipes da Estratégia Saúde da Família. Em 2020, a atenção primária de saúde do município acompanhou 1.439 gestantes de 10 a 19 anos, com pelo menos 1 consulta pré-natal realizada (16).

### Participantes

A população deste estudo foi composta por gestantes entre 10 e 19 anos que realizavam o pré-natal pelo Sistema Único de Saúde. Foram excluídas mulheres com condições obstétricas que impedissem a realização de atividades físicas na gestação atual, como placenta prévia, trabalho de parto prematuro, rotura prematura de membranas, pré-eclâmpsia ou hipertensão induzida pela gravidez, anemia grave, sangramento persistente no segundo ou terceiro trimestre, incompetência istmocervical, gestação múltipla com risco de parto prematuro, doença pulmonar restritiva e doença cardíaca hemodinamicamente significativa (12).

### Tamanho da amostra

Calculou-se o tamanho amostral levando em consideração o número de nascidos vivos entre adolescentes de 10 a 19 anos em Teresina no ano de 2020 e a prevalência de depressão pré-natal de 16% nas mulheres brasileiras (6). Assim, após aplicação do intervalo de confiança de 95%, erro de 5% e acréscimo de 10% para perdas, foi estimada amostra mínima de 182 participantes.

### Fonte de dados/controle de viés

A coleta de dados ocorreu em duas etapas. Primeiro, foram sorteadas unidades básicas de saúde de cada zona da cidade (Centro/Norte, Sul, Leste/Sudeste) até completar o tamanho amostral mínimo. Na segunda etapa, as adolescentes foram abordadas antes ou após a consulta pré-natal e convidadas a participar da pesquisa. Quando houve recusa ou não havia adolescentes realizando pré-natal, as instituições foram substituídas por outras na mesma zona. Nos casos em que as adolescentes não aceitaram ou não puderam participar, outra adolescente foi convidada em substituição. Todas as entrevistas foram conduzidas por pesquisador treinado e ocorreram na própria unidade de saúde onde a adolescente realizava o pré-natal. Realizou-se coleta piloto com 10 participantes para avaliar a compreensão das perguntas e, após ajustes, as respostas ao pré-teste foram descartadas. 

### Variáveis

Foram utilizados três instrumentos. O primeiro continha perguntas sobre variáveis: a) sociodemográficas: faixa etária (em anos: 10-14; 15-19); raça/cor da pele (branca; amarela; parda; preta; indígena); tipo de escola (pública; privada); escolaridade (<ensino fundamental; ≥ensino fundamental); escolaridade da mãe (não estudou; ensino fundamental incompleto ou completo; ensino médio incompleto ou completo; ensino superior incompleto e completo); evasão escolar (por causa da gravidez: sim; não); mora com mãe e/ou pai (sim; não); ocupação profissional (sim; não); situação conjugal (com parceiro; sem parceiro); b) comportamentais: consumo de álcool (na gestação: sim; não); consumo de cigarro (na gestação: sim; não); consumo de drogas ilícitas (na gestação: sim; não); transtorno de depressão (anterior à gravidez: sim; não); transtorno de ansiedade (anterior à gravidez: sim; não); ideações suicidas (anteriores à gravidez: sim; não); c) hábitos de atividade física: tipo de exercício físico (que costuma praticar: caminhada; natação; ciclismo; ginástica aeróbica; treinamento de força; yoga ou pilates; outro); prática de exercício físico recomendado por profissional de saúde (sim; não); prática de exercício físico com acompanhamento de profissional de educação física (sim; não); frequência de atividade física (vezes por semana: 1; 2-3; mais de 3; não se aplica); autopercepção de nível de atividade física (inativa; levemente ativa; ativa); d) obstétricas: número de gestações (incluindo a atual: 1; ≥2); número de abortos (0; ≥1); número de partos (0; ≥1); número de consultas pré-natais (<6; ≥6); gravidez planejada (sim; não); sentimento em relação à gestação não planejada (desejada; indesejada; não se aplica); e) relação conjugal: duração da parceria (<1 ano; >1 ano; não se aplica); comunicação com o parceiro (boa; ruim; não se aplica); brigas entre o casal (não; 01 vez/mês; >1 vez/mês; não se aplica). 

O segundo instrumento investigou o risco de depressão pré-natal, utilizando a Escala de Depressão Pós-Parto de Edimburgo, já validada no Brasil (17). É composto por 10 perguntas, utilizado para rastrear sintomas depressivos em mulheres grávidas ou no puerpério. Cada pergunta possui 4 alternativas pontuadas de 0 a 3 de acordo com intensidade dos sintomas, sendo que pontuações maiores ou iguais a 13 são consideradas como risco de desenvolver depressão (18). 

As informações sobre os hábitos de atividade física foram obtidas do Questionário de Atividade Física para Gestantes, direcionado para a população de mulheres grávidas e que investiga a relação entre a duração, a frequência, o tipo de atividade física e o gasto energético em 33 atividades (12 domésticas, 5 ocupacionais, 9 esportes/exercícios, 3 transportes/deslocamentos e 3 inatividades) nos últimos três meses. Cada questão possui valores em gastos energéticos equivalentes à sua intensidade e, para se obter a média de energia semanal, o consumo energético individual das atividades foi multiplicado pelo número de minutos em cada uma delas (19). Com esse resultado, foram avaliados: a) nível de atividade física: sedentário (valor inferior a 1,5); leve (1,5-3,0); moderado (3,0-6,0); vigoroso (superior a 6,0); b) nível de atividade física por domínio: tarefas domésticas; ocupacionais; cuidar de pessoas; esportes/exercícios; lazer; deslocamento. 

### Métodos estatísticos

Os dados foram analisados descritivamente por meio de frequências absolutas (n) e relativas (%) no programa Statistical Package for the Social Science. O teste do qui-quadrado de Pearson/teste exato de Fisher foi empregado para avaliar a associação entre o desfecho (depressão pré-natal) e a atividade física, além das outras variáveis independentes. Calculou-se razão de chances bruta (*odds ratio*, OR) e intervalos de confiança de 95% (IC95%). Todas as variáveis que mostraram p-valor<0,20 foram incluídas na análise multivariada por meio de regressão logística múltipla, com cálculo de razão de chances ajustada (*odds ratio*, OR) e IC95%. O nível de significância considerado foi de 5%. O modelo final de regressão foi ajustado pelas variáveis faixa etária, escolaridade, situação conjugal, gravidez não planejada, histórico de depressão e ideação suicida, pelo método Enter. 

## Resultados

Foram entrevistadas 182 adolescentes grávidas. A Tabela 1 mostra que a maioria tinha entre 15 e 19 anos (95,6%), raça/cor de pele parda (58,8%) e ensino fundamental (85,2%). A maior parte não residia com os pais (54,4%), possuía parceria conjugal (80,2%) e não relatou ocupação profissional (86,3%). Em relação à escolaridade materna, a maioria estudou até o ensino fundamental (40,7%). Todas as adolescentes estudaram em escola pública (100%) e quase um terço (30,2%) informou evasão escolar por causa da gestação.

**Tabela 1:** Características sociodemográficas das gestantes adolescentes. Teresina, 2023-2024 (n=182)

Variáveis	n (%)
**Faixa etária** (anos)	
10-14	8 (4,4)
15-19	174 (95,6)
**Raça/cor da pele**	
Branca	28 (15,4)
Preta	34 (18,7)
Amarela	10 (5,5)
Parda	107 (58,8)
Indígena	2 (1,1)
Não especificou	1 (0,5)
Escolaridade	
<Ensino fundamental	27 (14,8)
≥Ensino fundamental	155 (85,2)
**Mora com mãe e/ou pai**	
Sim	83 (45,6)
Não	99 (54,4)
**Situação conjugal**	
Com companheiro	146 (80,2)
Sem companheiro	36 (19,8)
**Escolaridade materna**	
Não estudou	6 (3,3)
Ensino fundamental	74 (40,7)
Ensino médio	73 (40,1)
Ensino superior	8 (4,4)
Não sabe/não quis especificar	21 (11,5)
**Tipo de escola**	
Pública	182 (100,0)
**Ocupação profissional**	
Sim	25 (13,7)
Não	157 (86,3)
**Evasão escolar**	
Sim	55 (30,2)
Não	127 (69,8)

A maioria estava grávida pela primeira vez (75,3%), sem partos (84,1%), sem abortos (87,4%) e com 6 ou mais consultas pré-natais (55,5%). O relato de gravidez não planejada foi o mais comum (80,2%), porém, entre estas, a maioria relatou que gestação foi desejada (89,7%). Houve predomínio de gestantes que estavam com um ano ou mais de relacionamento (74,7%) e que costumavam ter brigas entre o casal (62,3%). Quase todas informaram ter boa comunicação com o parceiro (97,3%). Durante a gestação, a maioria relatou não consumir bebidas alcoólicas (90,1%), não utilizar cigarro (97,8%) e drogas ilícitas (98,5%). Anterior à gravidez, houve relato de depressão (10,4%), ansiedade (41,8%) e ideações suicidas (20,9%). O risco de desenvolver depressão pré-natal foi de 31,3% (Tabela 2).

**Tabela 2:** Características obstétricas, comportamentais e da relação conjugal das gestantes adolescentes. Teresina, 2023-2024 (n=182)

Variáveis	n (%)
**Número de gestações**	
1	137 (75,3)
≥2	45 (24,7)
**Número de partos**	
0	153 (84,1)
≥1	29 (15,9)
**Número de abortos**	
0	159 (87,4)
≥1	23 (12,6)
**Número de consultas pré**-natais	
<6	81 (44,5)
≥6	101 (55,5)
**Gravidez planejada**	
Sim	36 (19,8)
Não	146 (80,2)
**Sentimento em relação à gravidez** (**não planejada**) (n=146)	
Desejada	131 (89,7)
Indesejada	15 (10,3)
**Duração da parceria** (n=146)	
Menos de um ano	37 (25,3)
Um ano ou mais	109 (74,7)
**Brigas entre o casal** (n=146)	
Não costuma brigar	55 (37,7)
Uma vez por mês	51 (34,9)
Mais de uma vez por mês	40 (27,4)
**Comunicação com o parceiro** (n=146)	
Boa	142 (97,3)
Ruim	4 (2,7)
**Uso de álcool**	
Sim	18 (9,9)
Não	164 (90,1)
**Uso de cigarro**	
Sim	4 (2,2)
Não	178 (97,8)
**Uso de drogas ilícitas**	
Sim	3 (1,5)
Não	179 (98,5)
**Transtorno de depressão**	
Sim	19 (10,4)
Não	163 (89,6)
**Transtorno de ansiedade**	
Sim	76 (41,8)
Não	106 (58,2)
**Ideação suicida**	
Sim	38 (20,9)
Não	144 (79,1)
**Risco de depressão pré-natal** (escore)	
<13	125 (68,7)
≥13	57 (31,3)

A maior parte delas não praticava exercício físico (67,6%). Entre as que praticavam, houve predomínio das que se exercitavam de 2 a 3 vezes por semana (57,6%), sem acompanhamento do profissional de educação física (91,5%) e sem recomendação de um profissional de saúde (78,0%), sendo a caminhada o exercício mais frequente (83,0%). Apesar de a maioria se considerar levemente ativa/ativa (73,6%), a pontuação obtida no Questionário de Atividade Física para Gestantes revelou que a maior parte foi classificada como sedentária (85,7%) (Tabela 3).

**Tabela 3:** Hábitos de atividade física das gestantes adolescentes. Teresina, 2023-2024 (n=182)

Variáveis	n (%)
**Prática de exercício físico**	
Sim	59 (32,4)
Não	123 (67,6)
**Frequência de exercício físico** (**vezes por semana**) (n=59)	
1	14 (23,7)
2 a 3	34 (57,6)
3 ou mais	11 (18,7)
**Exercício recomendado por profissional de saúde** (n=59)	
Sim	13 (22,0)
Não	46 (78,0)
**Exercício acompanhado por profissional de educação física** (n=59)	
Sim	5 (8,5)
Não	54 (91,5)
**Tipo de exercício** (n=59)^a^	
Caminhada	49 (83,0)
Ciclismo/ginástica aeróbica/dança	10 (16,9)
Treino de força/musculação/crossfit	7 (11,8)
Yoga/pilates	2 (3,4)
Natação	1 (1,7)
Outros	3 (5,0)
**Autopercepção do nível de atividade física**	
Sedentária	48 (26,4)
Levemente ativa	71 (39,0)
Ativa	63 (34,6)
**Nível de atividade física**	
Sedentário	156 (85,7)
Leve	25 (13,7)
Moderado	1 (0,6)

^a^Poderia ser marcado mais de uma alternativa.

O nível de atividade física por domínios pode ser observado na Tabela 4. O sedentarismo foi maior nos domínios esporte/exercício (100%), deslocamento (85,7%) e cuidar de pessoas (74,2%). O domínio que corresponde às tarefas domésticas apresentou maior gasto energético na classificação leve (49,0%) e o lazer na classificação moderado (18,7%). Já os domínios das atividades ocupacionais (3,9%) e das tarefas domésticas (1,1%) foram os únicos que tiveram participantes na classificação vigoroso.

**Tabela 4:** Nível de atividade física por domínios do Questionário de Atividade Física para Gestantes. Teresina, 2023-2024 (n=182)

Nível de atividade física	Sedentário n (%)	Leve n (%)	Moderado n (%)	Vigoroso n (%)
Tarefas domésticas	82 (45,0)	89 (49,0)	11 (6,0)	-
Cuidar de pessoas	135 (74,2)	23 (12,6)	22 (12,1)	2 (1,1)
Ocupacionais	129 (70,9)	27 (14,8)	19 (10,4)	7 (3,9)
Esporte/exercício	182 (100)	-	-	-
Deslocamento	156 (85,7)	22 (12,1)	4 (2,2)	-
Lazer	62 (34,1)	86 (47,2)	34 (18,7)	-

Na análise multivariada, após ajustes, identificou-se que as adolescentes grávidas que praticavam exercício físico (OR 0,22; IC95% 0,05; 0,94; p-valor 0,041) e que tinham gasto energético leve em atividade domésticas (OR 0,26; IC95% 0,07; 0,98; p-valor 0,047) exibiram menor chance de desenvolver depressão pré-natal. Ao contrário, ser sedentária no deslocamento (OR 2,40; IC95% 1,01; 5,68; p-valor 0,046) aumentou a chance de depressão pré-natal (Tabela 5).

**Tabela 5:** Razão de chances (*odds ratio*, OR) bruta e ajustada e intervalos de confiança de 95% (IC95%) da depressão pré-natal e atividade física. Teresina, 2023-2024

Variáveis	Depressão pré-natal			
	OR bruta (IC95%)	p-valor	OR ajustada^a^ (IC95%)	p-valor
**Prática de exercício físico**				
Sim	0,58 (0,28; 0,97)	0,026	0,22 (0,05; 0,94)	0,041
Não	1,00		1,00	
**Frequência semanal de exercício físico**				
1 vez	1,00		-	
2 a 3 vezes	0,90 (0,22; 3,60)	0,882	-	-
Mais de 3 vezes	0,25 (0,02; 2,65)	0,250	-	-
**Exercício recomendado por profissional de saúde**				
Sim	4,07 (1,08; 15,40)	0,031	1,87 (0,91; 3,87)	0,090
Não	1,00		1,00	
**Tipo de exercício**				
Caminhada	0,34 (0,07; 1,53)	0,159	0,29 (0,06; 1,37)	0,117
Caminhada e outro tipo	0,56 (0,09; 3,52)	0,538	0,50 (0,08; 3,21)	0,463
Outros	1,00		1,00	
**Autopercepção do nível de atividade física**				
Sedentária	1,20 (0,55; 2,63)	0,649	-	-
Levemente ativa	0,68 (0,32; 1,44)	0,538	-	-
Ativa	1,00			
**Nível de atividade física**				
Sedentária	1,03 (0,42; 2,53)	0,950	-	-
Leve/moderada	1,00			
**Nível de atividade física** (**tarefas domésticas**)				
Sedentária	0,39 (0,11; 1,38)	0,144	0,44 (0,12; 1,56)	0,206
Leve	0,33 (0,09; 1,16)	0,084	0,26 (0,07; 0,98)	0,047
Moderada	1,00		1,00	
**Nível de atividade física** (**cuidar de pessoas**)				
Sedentária	1,66 (0,58; 4,74)	0,346	1,60 (0,55; 4,64)	0,396
Leve	3,48 (0,97; 2,53)	0,056	3,00 (0,83; 11,24)	0,092
Moderada/vigorosa	1,00		1,00	
**Nível de atividade física** (ocupacional)				
Sedentária	2,70 (0,31; 23,14)	0,366	-	-
Leve	2,53 (0,26; 24,51)	0,424	-	-
Moderada	1,00			
Vigorosa	4,36 (0,43; 43,72)	0,210	-	-
**Nível de atividade física** (deslocamento)				
Sedentária	0,39 (0,17; 0,91)	0,027	2,40 (1,01; 5,68)	0,046
Leve/moderada	1,00		1,00	
**Nível de atividade física** (lazer)				
Sedentária	1,52 (0,62; 3,72)	0,364	-	-
Leve	0,87 (0,62; 3,72)	0,768	-	-
Moderada	1,00			

^a^Modelo ajustado por faixa etária, escolaridade, situação conjugal, gravidez não planejada, histórico de depressão, ideação suicida.

## Discussão

Os dados mostraram que cerca de um terço das adolescentes grávidas apresentou risco de desenvolver depressão pré-natal. A maioria não praticava exercícios físicos e, apesar de considerar ativa durante a gestação, a maior parte foi classificada como sedentária. Grande parte dos profissionais de saúde não recomendou prática de atividade física durante a gravidez e, entre as que se exercitavam, pequena parcela o fazia sob orientação de um profissional de educação física. A caminhada foi o tipo de exercício predominante, com maior gasto de tempo em tarefas domésticas e de lazer. Houve aumento da chance de depressão pré-natal nas que foram consideradas sedentárias no deslocamento. Por outro lado, praticar exercício físico e ter gasto energético leve em atividade domésticas determinou menor chance de desenvolver depressão pré-natal.

Pelo menos três limitações devem ser consideradas neste estudo. Primeiro, apesar do emprego de instrumentos validados, os autorrelatos podem ter sido afetados pelo viés de memória. Outra questão a ser mencionada é o curto período avaliado pelo Questionário (3 meses), sendo insuficiente para avaliar toda a gestação, levando à necessidade de estudos longitudinais. Por fim, as participantes eram mulheres grávidas que frequentavam serviços públicos, o que limita a generalização dos resultados. Por outro lado, um dos pontos relevantes deste estudo foi investigar a população de adolescentes grávidas, duplamente vulnerável e com poucas evidências, que apresentou elevados índices de inatividade física e de risco de depressão pré-natal. 

A prevalência de depressão pré-natal encontrada nesta pesquisa foi mais elevada que a observada em outros países. Cerca de 20% das mulheres sofrem com algum episódio de saúde mental durante a gravidez (1), com variação das taxas globais de depressão pré-natal de 15% a 65% e maior prevalência em países de baixa e média renda (20). No Brasil, a prevalência varia entre 11,4% e 26,8% (7,21). Entretanto, quando a amostra é composta somente por adolescentes grávidas, as taxas podem ser ainda mais elevadas, como as observadas em Pelotas, Rio Grande do Sul (29,2%) (22) e em Bandeirantes, Paraná (49,1%) (23).

As diferenças nos resultados podem ser atribuídas a variáveis socioeconômicas e culturais, como suporte social e níveis de estresse associados a diferentes contextos socioeconômicos (24). Disparidades socioeconômicas regionais, na educação, na saúde e no suporte social, podem influenciar esses resultados (7). Em regiões com menor acesso a serviços de saúde mental e maior incidência de pobreza, a prevalência de depressão pré-natal tende a ser mais alta (25). Além disso, o instrumento de avaliação empregado pode influenciar os resultados. Neste estudo, utilizou-se a Escala de Depressão Pós-Parto de Edimburgo, que geralmente evidencia níveis mais baixos de depressão (5).

A inatividade física foi predominante entre as gestantes adolescentes, sendo a maioria classificada como sedentária. Este achado é consistente com estudos que demonstram baixos níveis de atividade física entre mulheres grávidas globalmente (26,27). Apesar da escassez de informações específicas sobre a atividade física em gestantes adolescentes, os dados brasileiros evidenciam reduzido nível de atividade física durante a gestação nesse público (14,28). Em 2010, um estudo desenvolvido em Coari, Amazonas, verificou a percepção de sedentarismo entre adolescentes grávidas, identificando que 97% delas alegaram não praticar exercício durante a gravidez (28). 

Diversos fatores podem contribuir para a alta prevalência de inatividade física e sedentarismo nesta população. A falta de conhecimento e orientação sobre a importância da atividade física durante a gravidez pode levar essas jovens a subestimarem os benefícios do exercício regular (14,26,27). Barreiras socioeconômicas, como a falta de acesso a locais seguros para a prática de atividades físicas e a ausência de recursos financeiros para participar de programas de exercícios supervisionados, também desempenham papel significativo (29). Outro fator limitante é o medo de prejudicar a gestação, podendo ser exacerbado pela falta de orientação adequada por profissionais de saúde (30). A presença de sintomas depressivos e ansiosos também pode reduzir a motivação e a energia necessárias para a prática de atividades físicas (3,4). Combinados, esses fatores criam ambiente desfavorável para a adoção de estilo de vida ativo durante a gravidez entre adolescentes.

No atual estudo, a maior parte dos profissionais não recomendou a atividade física durante a gravidez, segundo o relato das adolescentes. Esse dado merece destaque por corroborar outras pesquisas que identificaram a baixa recomendação de exercício físico. Um estudo com 1.082 mulheres grávidas da África do Sul em 2019 constatou que 64,7% delas não recebeu qualquer aconselhamento sobre atividade física durante as consultas pré-natais (29). De forma semelhante, uma coorte com 118 gestantes portuguesas entre 2009 e 2011 observou que cerca de um terço dos profissionais de saúde não havia recomendado atividade física durante a gravidez e, dentre aqueles que o fizeram, a maioria recomendou apenas no segundo semestre de gestação (31).

A maioria das adolescentes que praticava atividade física o fazia de forma insuficiente e sem acompanhamento de profissionais de educação física, o que pode refletir dificuldades de acesso ou falta de conscientização sobre a importância desse suporte especializado. Apesar de se saber que o exercício físico regular promove diversos benefícios à saúde da grávida (11,26), especialistas alertam para cuidados necessários, como evitar atividades que envolvam contato físico, saltos, exercícios isométricos e mergulhos (12). Gestantes com certas condições de saúde, a exemplo de doença cardíaca ou respiratória, anemia grave, gestação múltipla, placenta prévia, pré-eclâmpsia e sangramento, não devem praticar exercício físico (12). O acompanhamento de um profissional de educação física é fundamental para elaborar programas de exercícios personalizados e seguros, prevenir lesões e complicações, além de fornecer suporte motivacional e psicológico. 

Outro aspecto que chamou atenção neste estudo foi que, mesmo entre as adolescentes que se consideravam levemente ativas e ativas, a maioria foi classificada como sedentária. Esse paradoxo pode ser atribuído a uma percepção errônea sobre o que constitui atividade física suficiente para alcançar benefícios à saúde, sugerindo que muitos indivíduos subestimam os níveis de atividade necessários para manter a saúde mental e física (11,27). Gestantes frequentemente consideram suas atividades diárias como suficientes para se manterem fisicamente ativas, apesar de não atingirem os níveis recomendados de atividade física (26,29). O tipo de exercício físico mais praticado pelas gestantes adolescentes foi a caminhada, com predomínio de sessões de 2 a 3 vezes por semana. A recorrência dessa prática é, possivelmente, devido à acessibilidade e à facilidade de sua realização (26,27,29). Entretanto, a falta de variedade e a baixa intensidade das atividades se refletem no alto índice de sedentarismo identificado pelo Questionário de Atividade Física para Gestantes nesta pesquisa.

A maior parte do tempo semanal das gestantes desta pesquisa foi gasto em atividades de lazer e domésticas, com maior prevalência de sedentarismo nos domínios esporte/exercício, deslocamento e cuidar de pessoas. De forma análoga, uma investigação em 2019 com 1.279 gestantes de Montes Claros, Minas Gerais, identificou que nas categorias cuidar de pessoas, esportes e deslocamento, respectivamente, 89,0%, 93,1% e 93,2% das entrevistadas foram classificadas como inativas fisicamente (32). Esses números refletem um estilo de vida que carece de movimentação física regular, sobretudo em atividades físicas estruturadas ou esportivas, o que é preocupante, pois a prática regular de exercícios durante a gravidez está associada a benefícios significativos tanto para a mulher quanto para o feto (11).

Adolescentes classificadas como sedentárias no deslocamento apresentaram maior chance de desenvolver depressão pré-natal em comparação àquelas com níveis de atividade física leve ou moderado nesse domínio. Este resultado é consistente com outras pesquisas que demonstraram que o deslocamento ativo, como caminhar ou andar de bicicleta, pode proporcionar benefícios psicológicos significativos, incluindo a redução do risco de depressão, provavelmente por exposição ao ambiente externo e à interação social (33,34). Já as adolescentes que praticavam exercício físico e aquelas com gasto energético leve em atividades domésticas apresentaram menor chance de desenvolver depressão pré-natal. Por um lado, em virtude de ser estressante principalmente para as mulheres, a atividade física doméstica pode não ter efeito ou mesmo aumentar o risco de depressão (35). Por outro lado, mesmo em níveis leves, esse tipo de atividade física tem efeito protetor contra a depressão durante a gravidez (36,37).

Mesmo ainda existindo dúvidas sobre qual tipo de atividade física é mais efetiva para mitigar os efeitos da depressão pré-natal e pós-parto, a Organização Mundial da Saúde recomenda que todas as grávidas sem contraindicação realizem pelo menos 150 minutos semanais de exercícios aeróbicos de intensidade moderada, associado com treinamento de força e atividades de relaxamento (38). Tanto exercícios aeróbicos como exercícios em grupo, atividades em ambientes aquáticos e aquelas de escolha da grávida podem aliviar os sintomas da depressão pré-natal (36,37,39). No entanto, no final da gravidez as mulheres tendem a se sentir fisicamente cansadas e atividades físicas leves, como meditação e relaxamento corporal, também exibem impacto positivo sobre a saúde mental das grávidas (37,39).

Considerando a prática de atividade física como tendo efeito positivo sobre a ocorrência da depressão pré-natal, os resultados do presente estudo podem corroborar com a implementação de intervenções que incentivem a prática de atividade física entre adolescentes. Pesquisas futuras deveriam explorar os tipos, a intensidade e a duração da atividade física mais adequados e eficazes para níveis de intensidade da depressão. Um dos desafios diz respeito à necessidade de intervenções personalizadas que levem em conta não apenas a recomendação de profissionais de saúde, mas também as preferências e o contexto individual das adolescentes para promover a atividade física. Considerando a vulnerabilidade dessa população, estratégias de promoção da saúde que integrem suporte emocional e motivacional podem ser especialmente benéficas. Do ponto de vista da saúde pública, a inserção e a atuação do profissional de educação física na atenção primária ampliam a oferta de promoção da saúde multiprofissional, por meio de práticas corporais e de atividade física sistematizada. É possível que tais ações resultem na redução da elevada prevalência de depressão pré-natal e dos desfechos negativos a ela relacionados.

## Data Availability

O acesso ao banco de dados e os códigos de análise, métodos e outros materiais utilizados na pesquisa poderão ser solicitados ao autor coordenador da pesquisa ou ao autor correspondente.
